# Validity and reliability of the Generalized Anxiety Disorder-7 (GAD-7) among university students of Bangladesh

**DOI:** 10.1371/journal.pone.0261590

**Published:** 2021-12-16

**Authors:** Tahia Anan Dhira, Mahir A. Rahman, Abdur Razzaque Sarker, Jeenat Mehareen

**Affiliations:** 1 Department of Economics, University of Dhaka, Dhaka, Bangladesh; 2 Bangladesh Institute of Development Studies, Dhaka, Bangladesh; 3 Department of Economics, East West University, Dhaka, Bangladesh; Universita degli Studi Europea di Roma, ITALY

## Abstract

This study investigated the reliability and factorial validity of General Anxiety Disorder-7 (GAD-7) in the context of university students in Bangladesh. The research aimed to assess whether the original one-dimensional model or a model containing both somatic and cognitive-emotional factors is appropriate. A repeated cross-sectional survey design based on convenience sampling was used to collect data from 677 university students. The factor structure of the GAD-7 was assessed by exploratory factor analysis (EFA) and confirmatory factor analysis (CFA), and its convergent validity was determined by investigating its correlations with Patient Health Questionnaire-9 (PHQ-9) and Patient Health Questionnaire Anxiety-Depression Scale (PHQ-ADS). Results showed excellent reliability of GAD-7 as measured by Cronbach’s α. CFA suggested that a modified one-factor model is appropriate for the sample. This model provided high values of comparative fit index (CFI), goodness of fit index (GFI), and Tucker Lewis Index (TLI), low value of standardized root mean square residual (SRMR) and a non-significant root mean square error of approximation (RMSEA). Correlation between GAD-7 and PHQ-9 was 0.751 and 0.934 between GAD-7 and PHQ-ADS. Overall, the study provided support for modified unidimensional structure for GAD-7 and showed high internal consistency along with good convergent validity.

## Introduction

Generalized Anxiety Disorder (GAD) belongs to a group of mental health conditions collectively known as anxiety disorders, which also includes panic disorder, phobias, social anxiety disorder, Obsessive-Compulsive Disorder (OCD) and Post-Traumatic Stress Disorder (PTSD) [1]. GAD reflects the fundamental characteristics of all emotional disorders and is used to diagnose subsequent occurrence of any other anxiety disorders [2, 3]. The definition of GAD emerged from the third edition of the Diagnostic and Statistical Manual of Mental Disorders (DSM-III) where it was characterized as a residual diagnosis and only attributed to patients who did not meet diagnostic criteria for any other anxiety disorders [3–5]. After undergoing several substantial revisions, GAD was characterized as an independent anxiety disorder with diagnostic description. In the Diagnostic and Statistical Manual of Mental Disorders, Fifth Edition (DSM-5), GAD has been stated to include both psychological symptoms such as persistent worrying and overthinking, difficulty handling uncertainty, restlessness, indecisiveness, etc. as well as physiological symptoms such as fatigue, sleeplessness, muscle tension, nervousness, nausea, irritability, etc. [6, 7]. Findings from general population surveys of approximately 150000 adults in 26 countries indicated that DSM-5 GAD had a combined lifetime prevalence of 3.7%, 12-month prevalence of 1.8%, and 30-day prevalence of 0.8% [8].

Among South Asian countries, as of 2017, the prevalence and severity of anxiety disorder is the highest in Bangladesh. 4.4% of the Bangladeshi population is estimated to have this disorder and it contributes to 4% of the total years lived with disability (YLD) [9, 10]. Specifically, a study estimated prevalence of GAD to be around 37.3% among individuals of Bangladesh aged between 13 to 63 years [11]. The prevalence of symptoms of anxiety was particularly high in case of university students [12, 13]. A recent study conducted by Faisal et al. (2021) indicated that approximately 40% of Bangladeshi students suffered from symptoms of moderate to severe GAD, a rate which is higher than those reported in the studies conducted on general people [14, 15]. University students go through significant changes in their emotional (e.g., loneliness, personal autonomy) and physical environment (e.g., transition from college to university) [16]. They also deal with the pressure of competition for good grades, carefully plan for future career, participate in extracurricular activities and are more involved in family matters as an adult [17]. Therefore, it is not unexpected for students to be more vulnerable to symptoms of anxiety disorders.

Despite high prevalence and potentially severe consequences including decreased quality of life, multiple somatic complaints, maladaptive personality traits, and increased mortality due to suicide, cardiovascular, and cerebrovascular causes, GAD has been largely ignored as a mental health condition [18–21]. One important barrier to effective treatment has been inaccurate or no assessment. People who suffer from anxiety disorders are often under-diagnosed and/or treated inadequately [17]. To overcome the hurdles, there is a need for a reliable, valid, and relatively brief screening tool for anxiety disorders, particularly for university students. The seven-item Generalized Anxiety Disorder scale (GAD-7) was developed with the purpose of screening for, and assessing the severity of GAD symptoms in both clinical and research contexts [22]. Conducted on a large primary care sample, the first validation study of GAD-7 found good reliability, as well as criterion, construct, factorial, and procedural validity. Subsequently, the psychometric properties of GAD-7 have been evaluated in different sample of patients, including other primary care samples [23, 24], psychiatric patients [25–27], people with addiction [28], pregnant woman [29, 30], and outpatients [29–31]. GAD-7 has also shown strong psychometric properties in different populations, such as general adults [32], elderly [33, 34] as well as people from different culture and countries [35–39]. In academic setting, validation was performed among Korean university students [40], Portuguese college students [41] and Saudi university male students [42]. In the context of Bangladesh, GAD-7 has been widely used in various studies as a screening tool for anxiety symptoms among adults and university students [11–13]. Moreover, Faisal et al. (2021) explored the psychometric properties of the Bengali version of the GAD-7; however, the main focus of the paper was to assess the impact of the COVID-19 pandemic on anxiety, depressive symptoms and mental health status of university students [14]. Thus, a detailed assessment of factorial validity of GAD-7 focusing on Bangladeshi students has not been carried out yet.

Regardless of adequate internal consistency and moderate to strong correlation with other comorbid disorders (depression, worry, etc.), findings regarding the factor structure of GAD-7 have not been consistent [43, 44]. Some studies showed a unidimensional factor structure [37, 41, 45–47]; which aligns with the result of the original validation study. Contrastingly, others indicated a two-dimensional factor structure [25, 27]. Given the lack of consensus regarding the factor structure of GAD-7 in different contexts, a comprehensive validation study for Bangladeshi university students may be beneficial for an easy and cost-effective screening of anxiety disorders in this population.

Against this backdrop, we conducted this study with the objective of investigating the reliability and factorial validity of GAD-7 in the context of university students in Bangladesh. To this effect, the research mainly aimed to assess whether the original one-dimensional model or a model containing both somatic and cognitive-emotional factors is appropriate for both public and private university students. We further examined the convergent validity of GAD-7 with other relevant measures of mental health conditions, namely Patient Health Questionnaire-9 (PHQ-9) and Patient Health Questionnaire Anxiety-Depression Scale (PHQ-ADS). Finally, we examined the mean GAD-7 score of the students across different demographic and socioeconomic correlates. We expect that the study will contribute to the growing body of literature pertaining to validation studies assessing symptoms of anxiety disorders in university students.

## Methods

### Procedure and sampling

A repeated cross-sectional survey design was used to collect responses among the university students of Bangladesh. Data was collected in two waves: July 18-July 31, 2020 and February 10-February 22, 2021; the survey Administration software Google Form [48]. Snowball sampling strategy was utilized to capture both public and private university students. To be eligible for the study, the participants had to meet the following criteria: (a) be willing to participate in the study; (b) be enrolled in any public or private university in Bangladesh; (c) have internet access; and (d) be able to read, write, and comprehend the English questionnaire.

Bangladesh has roughly 1.3 million students currently pursuing higher education in 47 public and 107 private universities [49, 50]. Considering this population, we calculated the sample size based on the formula:

$$n=\frac{{\text{z}}^{2}\text{p}\left(1-\text{p}\right)}{{\text{e}}^{2}}$$

where, *n* is the sample size, z is the selected critical value of the desired confidence level, p is the estimated proportion of an attribute that is present in the population, and e is the desired level of precision. Using 5% margin of error, 99% confidence level, and 50% response distribution, the sample size was estimated to be 666.

The questionnaire was circulated among two public and three private university students. Students from these universities were most likely to have access to a suitable internet connection and also use English as a mode of learning. Therefore, it was convenient for us to reach them through social media platforms while keeping the questionnaire in its original form. The questionnaire (Google Form link) was initially shared with faculty members of those selected universities, and they were asked to distribute the questionnaire in their respective classrooms either via e-mail or through any course material sharing platform that they were using for communication. We also asked the faculty members to encourage the students to pass on the survey link among their classmates to ensure maximum data collection. The final collection of data had a sample of 677 participants studying at different levels of university.

### Description of GAD-7

In order to assess the presence and severity of GAD, a self- administered seven-item instrument GAD-7 is used as a screening tool [22, 23, 51]. Its items describe the prominent diagnostic features of the original DSM-IV diagnostic criteria for generalized anxiety disorder [52]. In the assessment, participants are asked how often during the last two weeks they have encountered anxiety symptoms like feeling nervous, trouble relaxing etc. Response options for each item range from 0 to 3 on a 4-point Likert-scale (0 = not at all, 1 = several days, 2 = more than half the days and 3 = nearly every day). Adding the scores of all seven items provide the GAD-7 total score ranging from 0 to 21. Several validation studies have detected cut-points of ≥5, ≥10 and ≥15 based on receiver operating characteristics analysis for GAD-7, standing for mild, moderate and severe anxiety levels, respectively [52].

PHQ-9 and PHQ-ADS scales were also used to test for convergent validity of GAD-7. The PHQ-9 assesses the frequency and severity of symptoms of depression using nine 4-point Likert-scaled items ranging from 0 (not at all) to 3 (nearly every day) [53]. A total score ranging from 0 to 27 is obtained by summing across all items. The total score can be categorized at a cutoff of 10 to differentiate between minimal/mild versus moderate/severe depression. On the other hand, the PHQ-ADS is a composite measure that assess the overall burden of anxiety and depressive symptoms (mental distress) while combining the sum of the PHQ-9 and GAD-7 scores [54]. Thus, the scale can range from 0 to 48, with higher scores indicating higher levels of depression and anxiety symptomatology. Cut points of 10, 20, and 30 on the PHQ-ADS can be considered as thresholds of mild, moderate, and severe distress symptoms, respectively.

### Statistical analysis

Characteristics of the items were examined by exploring item mean score, item-intercorrelations and corrected item-total correlations. Internal consistency and reliability were assessed by using Cronbach’s α.

To analyze construct validity of GAD-7, maximum likelihood Exploratory Factor Analysis (EFA) was performed. For applicability purpose, Bartlett Test of Sphericity and Kaiser-Meyer-Olkin (KMO) measure of sampling adequacy were assessed prior to executing Principal Component Factoring (PCF). The number of factors to be retained by PCF was evaluated by (i) using eigenvalue of more than 1, (ii) visually inspecting the scree plot, (iii) considering the items which had factor loading of at least 0.40 per factor and (iv) were interpretable [55].

Based on the results from the EFA, confirmatory factor analysis (CFA) was performed with structural equation model (SEM). CFA is a multivariate procedure used to validate the factor structure of a set of observed variables [56].To determine the areas of misfit in the model suggested by EFA, modification indices were inspected. Next, the original model and the modified model were compared using several model fit indices and their criteria, including (i) the chi-square (*χ*^2^) and its degrees of freedom (df), (ii) root mean square error of approximation (RMSEA) and its 90% confidence interval, (iii) comparative fit index (CFI), (iv) goodness of fit index (GFI), (v) Tucker Lewis Index (TLI) and (vi) standardized root mean square residual (SRMR) (Table 3). RMSEA values of less than or equal to 0.05 represents close fit, while values between 0.05 to 0.08 are considered acceptable fit [57, 58]. Likewise, based on previous studies, GFI values greater than 0.9 indicate good fit [59]. CFI [59] and TLI [60] are incremental fit indices and values of greater than or equal to 0.95 of these indices indicate very good fit [60], while values of 0.90 or above are considered acceptable fit. Additionally, SRMR values up to 0.05 indicate close-fit, whereas values between 0.05 to 0.10 suggest acceptable fit [60].

To assess convergent validity of the GAD-7, the associations between GAD-7 and PHQ-9 and PHQ-ADS were examined using Pearson’s correlation (r) and its significance. Relationship between GAD-7 score and socio-demographic variables was also studied using t-test and analysis of variance. Data cleaning, validation, and all statistical analysis were performed using Stata/IC 16.1 (StataCorp, College Station, TX, USA).

### Ethical considerations

Ethical permission for data collection was taken from respective faculty and department heads of the universities where the questionnaire was distributed. Before responding anonymously to the online survey, all participants voluntarily gave their informed consent to participate in the study. In the consent form, participants were provided with information concerning the purpose, procedure and nature of the study, the option to take part as well as the right to revoke their data at any point of the study. The research is approved by the Department of Economics, East West University and procedures of this study complied with the provisions of the Declaration of Helsinki (1989) regarding research on human participants.

## Results

### Distribution of socio-demographic variables

Among the 677 participants included in the study, 51.40% were male and 48.60% were female. The mean age of the participants was 21.11 years. Almost two-third of the sample (65.19%) were made up of public university students and 45.64% of the students were studying in first year. The distribution of the main socio-demographic characteristics is presented in Table 1.

**Table 1:** Socio-demographic variables.

Variables	Categories	*n*	% in the sample
**Gender**	Male	348	51.40
	Female	329	48.60
**Age** ^**a**^	18–22 years	550	81.24
	23–27 years	127	18.76
**Marital Status**	Married	8	1.19
	Single	661	97.93
	Others	6	0.89
**Type of University**	Public University	440	65.19
	Private University	235	34.81
**Level of Education**	First year	309	45.64
	Second year	98	14.48
	Third year	131	19.35
	Fourth year	95	14.03
	Masters	44	6.50
**Principal Source of Income**	Government Service Holder	189	27.92
	Agricultural wage labor	35	5.17
	Organized Trade/Business	173	25.55
	Pension/ Rent	87	12.85
	Private Service Holder	181	26.74
	Others	12	1.77
**Student employment**	Yes	102	15.07
	No	575	84.93
**Family monthly income**	<25,000 BDT	154	22.75
	25,000–54,999 BDT	250	36.93
	55,000–99,999 BDT	174	25.70
	> = 1,00,000 BDT	99	14.62
**Family Size**	< = 4 members	340	50.22
	>4 members	337	49.78
**Joint Family**	Yes	141	20.83
	No	536	79.17
**Domestic violence in family**	Yes	96	14.18
	No	581	85.82
**Whether the student is the victim of domestic violence**	Yes	65	67.71
	No	31	32.29
**Majority of time spent**	Alone	191	28.21
	With family	409	60.41
	With friends	66	9.75
	With pets	11	1.62

^a^ Mean (SD): Age, 21.11 (0.064), CI (20.99–21.24).

BDT = Bangladeshi Taka.

### Item characteristics and reliability

Item characteristics are summarized in Table 2. The mean (SD) value of GAD-7 scale is 9.87 (6.05) and mean value of the items ranges from 1.04 to 1.65. Corrected item-total correlations (i.e. correlation between an item and the scale that is formed by all other items) are found to be between 0.587 to 0.784 indicating maximum performance tests [61, 62].

**Table 2:** Characteristics of items and total GAD-7 scale.

	I	II	III	IV	V	VI
GAD-7 Items	Mean	SD	Item Total Correlation ^a^	Corrected Item-Total Correlation ^b^	Factor Loadings	Cronbach’s α
	(95% CI)					
1. Feeling nervous, anxious or on edge?	1.42	1.08	0.807	0.729	0.814	0.875
	(1.34–1.50)					
2. Not being able to stop or control worrying?	1.53	1.15	0.842	0.771	0.848	0.870
	(1.44–1.61)					
3. Worrying too much about different things?	1.65	1.12	0.850	0.784	0.857	0.868
	(1.57–1.73)					
4. Trouble relaxing?	1.30	1.09	0.756	0.661	0.754	0.883
	(1.23–1.38)					
5. Being so restless that it is hard to sit still?	1.04	1.07	0.697	0.587	0.686	0.891
	(0.96–1.12)					
6. Becoming easily annoyed or irritable?	1.38	1.09	0.755	0.660	0.753	0.883
	(1.30–1.47)					
7. Feeling afraid as if something awful might happen?	1.55	1.15	0.767	0.669	0.762	0.882
	(1.46–1.63)					
GAD-7 Total Score	9.87	6.05	___	___	___	0.895
	(9.41–10.33)					

^a^ Correlation between the item and the total score from the scale when the item itself is included in the total.

^b^ Correlation between the item and the total score from the scale when the item itself is not included in the total.

CI = Confidence Interval; SD = Standard Deviation.

The reliability coefficient Cronbach’s α for the overall GAD-7 scale is 0.895, which is greater than the recommended value of 0.80 suggesting excellent reliability [63]. Besides, there is no alpha for any of the items which is greater than the overall alpha, suggesting excellent reliability even if an item is deleted (Column VI, Table 2). All intercorrelation between GAD-7 items were significant at *p*<0.01 (S1 Table).

### Construct validity

**Fig 1:**
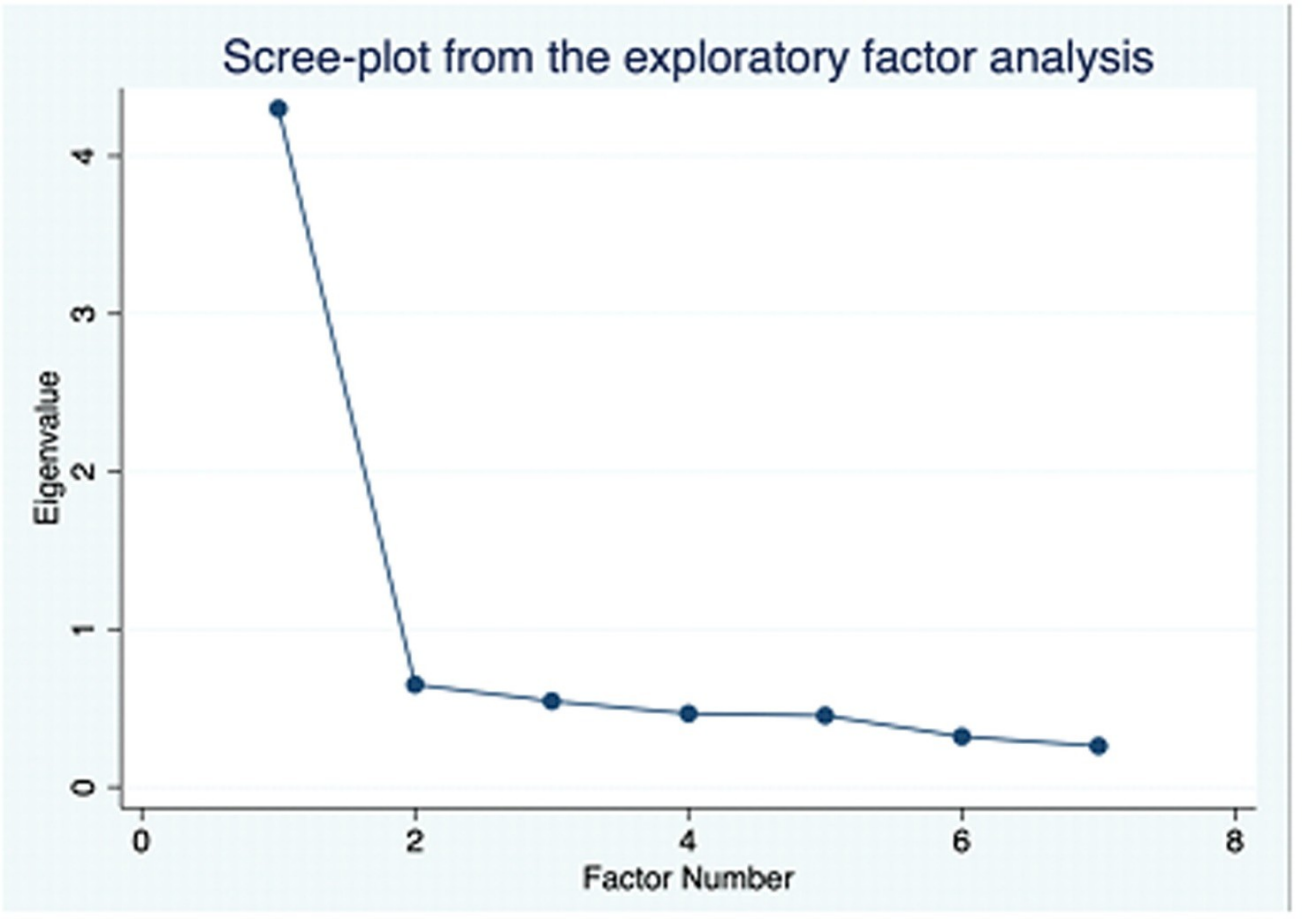
Scree-plot of GAD-7.

Construct validity of GAD-7 was tested with exploratory and confirmatory factor analysis. Applicability of factor analysis was tested using KMO and Bartlett Test of Sphericity. The KMO coefficient is 0.915 which surpasses the recommended value of 0.6, while Bartlett Test of Sphericity is found statistically significant (*χ*^*2*^ = 2425.95, df = 21, p<0.01), indicating a factor analysis can be conducted in this case [64]. As a first step, we conducted an exploratory factor analysis to investigate the factor structure of GAD-7. Only one factor was extracted based on eigenvalue and scree plot inspection which alone accounts for 61.40% of the total variance of GAD-7 (S2 Table and Fig 1). All the items of GAD-7 had statistically significant loadings with values greater than 0.5 (Table 2). Therefore, all seven items of the measure are important to interpret.

**Fig 2:**
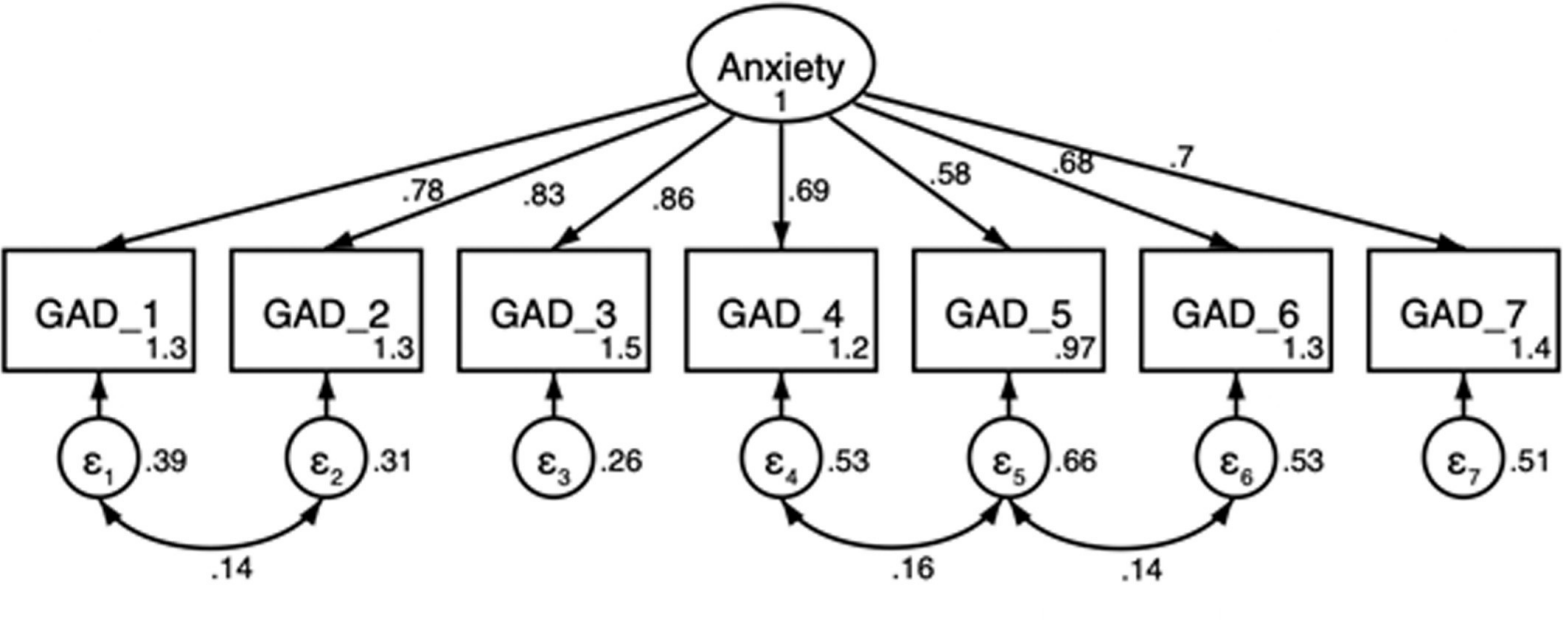
Confirmatory factor analysis path diagram for modified one-factor model of GAD-7 factors.

Based on the result of EFA, a one-factor model was analyzed for the CFA. The results do not confirm adequate fit criteria for the initial one-factor model (*χ*^*2*^ = 61.43, df = 14; *p*-value<0.01; CFI = 0.980; GFI = 0.975; TLI = 0.971; RMSEA = 0.071 (90% CI, 0.053–0.089, *p*<0.05); SRMR = 0.028) (Table 3). While CFI, GFI and TLI have values greater than the recommended threshold (0.950) and SRMR is indicates close fit (<0.05), the chi-square value is significant at *p*<0.01 suggesting poor fit of the model. Besides, chi-square provides inflated value when sample size is large and does not work well when in cases where sample size is small, and the underlying distribution may be non-normal [65]. Moreover, the RMSEA value is above the recommended upper limit of 0.05 and is statistically significant. Therefore, as a next step, we tested whether GAD-7 fits the two-factor structure (cognitive and somatic) for our sample. The cognitive factor comprises of item 1, 2, 3 and 7 whereas the somatic factor comprises of items 4, 5 and 6. While the two-factor model performed better in all fit indices compared to the one-factor model (Table 3), the covariance between the two factors was extremely high (0.931). In cases where two factors are highly overlapping, because of parsimony concerns, it is customary to inspect modification indices to decide whether a modified one-factor structure provides a reasonable fit [65]. Based on examination of modification indices, the error terms of item-1 and item-2, item-4 and item-5 and item-5 and item-6 were allowed to covary to improve the goodness of fit of the model. The modified model then provided a non-significant chi-square, higher values of CFI, GFI and TLI (0.986, 0.981 and 0.978, respectively) and lower value of SRMR (0.024) as well as a non-significant RMSEA with a lower value (0.061). All factor loadings and error covariances are statistically significant (*p*<0.01), suggesting that the indicator variables are significantly related to their respective factor. Confirmatory factor analysis path diagram is represented in Fig 2 and the fit indices are shown in Table 3.

**Table 3:** Goodness of fit indices for the GAD-7 -item factor models (*n* = 677).

Model	k	*χ* ^2^	df	CFI	GFI	TLI	RMSEA (90% CI)	SRMR
One-factor model	7	61.431**	14	0.980	0.975	0.971	0.071*(0.053–0.089)	0.028
Two-factor model	7	44.721	13	0.987	0.982	0.978	0.060 (0.041–0.080)	0.021
Modified one-factor model	7	26.105	11	0.994	0.989	0.988	0.045(0.023–0.068)	0.017

*p<0.05,

** *p*<0.01.

k = number of items; df = degrees of freedom; CFI = comparative fit index; GFI = goodness of fit index; TLI = Tucker-Lewis index; RMSEA = root mean squared error of approximation; SRMR = standardized root mean residual; CI = Confidence Interval.

### GAD-7 scores across socio-demographic characteristics

Table 4 shows the mean score of GAD-7 index across different socio-demographic variables. From the table, it is observed that female students have higher mean GAD-7 scores compared to males. In counter to private university students, mean GAD-7 score for public university students was also significantly higher. Moreover, mean GAD-7 scores are significantly different among students enrolled in different educational levels in university ranging from first year to fourth year, where higher levels students had higher average GAD-7 scores. Furthermore, students who faced domestic violence in the family and spent time alone reported significantly higher mean GAD-7 scores. Of note, there was no difference in mean GAD-7 scores among students with different age group, family income and family size.

**Table 4:** Association of GAD-7 scores with socio-demographic characteristics (*n* = 677).

Variables	Categories	GAD-7 Mean Score (SD)	*t/F*	*p*-value
**Gender**	Male	8.73 (5.91)	5.133	<0.01**
	Female	11.08 (5.98)		
**Age**	18–22 years	9.83 (5.89)	-0.333	0.739
	23–27 years	10.03 (6.75)		
**Marital Status**	Married	10.25 (6.65)	0.48	0.62
	Single	9.88 (6.03)		
	Others	7.5 (7.42)		
**Type of University**	Public University	10.46 (6.15)	-3.559	<0.01**
	Private University	8.74 (5.73)		
**Level of Education**	First year	8.94 (5.74)	5.03	<0.01**
	Second year	9.69 (5.76)		
	Third year	11.60 (6.22)		
	Fourth year	10.68 (6.45)		
	Masters	9.86 (6.36)		
**Principal Source of Income**	Government Service Holder	9.60 (5.93)	1.92	0.088
	Agricultural wage labour	9.43 (6.25)		
	Organized Trade/Business	8.97 (5.79)		
	Pension/ Rent	10.43 (6.33)		
	Private Service Holder	10.81 (6.22)		
	Others	10.17 (5.22)		
**Student employment**	Yes	9.20 (5.68)	1.22	0.223
	No	9.99 (6.12)		
**Family monthly income**	<25,000 BDT	9.31 (5.89)	0.86	0.463
	25,000–54,999 BDT	9.88 (5.89)		
	55,000–99,999 BDT	10.38 (5.96)		
	> = 1,00,000 BDT	9.84 (6.85)		
**Family Size**	< = 4 members	10.06 (6.01)	-0.815	0.416
	>4 members	9.68 (6.12)		
**Joint Family**	Yes	8.67 (5.93)	2.601	<0.05*
	No	10.18 (6.05)		
**Domestic violence in family**	Yes	11.48 (5.92)	-2.825	<0.01**
	No	9.60 (6.04)		
**Majority of time spent**	Alone	12.39 (5.67)	17.81	<0.01**
	With family	8.65 (5.82)		
	With friends	10.11 (6.60)		
	With pets	10.09 (4.81)		

^a^ Group differences were performed using *t-*test and analysis of variance.

*p<0.05,

** *p*<0.01.

SD = Standard Deviation, BDT = Bangladeshi Taka.

### Convergent validity

Pearson correlation coefficients were calculated to examine convergent validity of the GAD-7 with other measures (PHQ-9 and PHQ-ADS). Scores on GAD-7 scale were highly and positively correlated with the scores of PHQ-9 and PHQ-ADS. Correlation between GAD-7 and PHQ-9 is 0.751 and between GAD-7 and PHQ-ADS is 0.934. Both correlations are statistically significant (*p*<0.01) (S1 Table).

## Discussion

GAD-7 has been used to detect symptoms of anxiety disorders in various settings and across diverse populations, beyond its original application in primary-care settings. Therefore, evaluating the psychometric properties of the scale is necessary. Furthermore, the paucity of studies conducted on vulnerable groups such as university students also necessitates a contribution to the existing gap in the literature. In this context, our study examined the psychometric properties of the GAD-7 with a sample of university students in Bangladesh, using EFA and CFA.

In the present study, internal consistency of the scale was excellent, reflected by the overall Cronbach’s α of 0.895. The original validation study conducted on adult patients in primary care clinics in USA reported a Cronbach’s α score of 0.92 [22]. Other clinical and non-clinical studies conducted in Korea, Portugal, United States, Iran, Germany and, Peru have similarly found excellent Cronbach’s α coefficient [30, 40, 41, 46, 47, 66] which show good internal consistency of GAD-7 scale across different populations. Specifically, surveys conducted on university students of Korea and college students of Portugal found on Cronbach’s α coefficient to be 0.91 and 0.88 respectively, revealing excellent internal consistency. [40, 41]. In Bangladesh, Faisal et al. (2021) found good internal consistency of GAD-7 (Cronbach’s α = 0.87) in a study on university students [14]. We tested the convergent validity of GAD-7 with two other scales, PHQ-9 and PHQ-ADS. Correlation of GAD-7 with both PHQ-9 and PHQ-ADS were strong, with the coefficients statistically significant and greater than 0.75, suggesting good convergent validity. Studies have found comorbidity of GAD with depressive disorders [67, 68] and it has been identified as a predictor of mood disorders [69]. Hettema (2008) noted that GAD has undergone a series of revisions in its diagnostic criteria that has moved it, nosologically, away from its original affiliation with panic disorder (PD) and closer to major depressive disorder (MDD). Accordingly, he found a strong overlap between GAD and MDD based upon familial/genetic, childhood environment, personality trait, and demographic data. Schoevers et al. (2005) studied 2,173 community-living elderly persons and found that GAD often coexists with depression. Therefore, relationship between scales measuring symptoms of anxiety and depression is not unexpected. The study on Korean university students has also found good convergent validity of GAD-7 with PHQ-9, with a correlation coefficient of 0.68 [40]. The study conducted on Portuguese college students found strong correlation (r>0.8) of GAD-7 with HADS-A and HADS-D scale (Hospital Anxiety and Depression Scale) [41]. Other studies have also found strong evidence of convergence validity of GAD-7 with similar psychometric instruments in different settings [25, 47]. All these results signify the usefulness of GAD-7 scale as a screening tool for anxiety disorder among university students in different countries.

The unidimensional model proposed by EFA showed a marginal fit to our context. Hence, the original model was amended using the examination of modification indices. Dependency between the error terms of item-1 (‘Feeling nervous, anxious or on edge’) and 2 (‘Not being able to stop or control worrying’), 4 (‘Trouble relaxing’) and 5 (‘Being so restless that it is hard to sit still’) and 5 and 6 (‘Becoming easily annoyed or irritable’) upgraded the fitness of the model. Our modified one-factor model was partially similar to that for Korean university students [40] and for Portuguese college students [41]. While these studies found that there is covariance between error terms of item 4 and item 5 as well as item 5 and item 6, error terms of item 1 and item 2 were not found to covary. Lowe et al. (2008) and Kertz et al. (2013) have also confirmed the modified one-factor structure of the GAD-7 scale in samples of general population and patients enrolled in a partial hospital program, respectively [27, 47]. However, a study on Saudi university male students found support for the original one-factor structure of GAD-7 [42], whereas the two-factor structure of GAD-7 was supported by some other studies [25, 70]. In consistence with results from most of the studies conducted on samples of students, the unidimensional structure of GAD-7 also fits well in our study. [40, 41].

Aside from the issues discussed above, the mean scores obtained from GAD-7 across different socio-demographic characteristics were in line with existing literature on the university students around the globe [71–73]. In terms of gender, higher GAD-7 scores were associated with female students. Such finding is not unexpected as according to several studies, gender difference in incidence of psychological disorders is pervasive [31, 74, 75], including university students in Dhaka, Bangladesh [76]. In case of level of education, we observe that students enrolled in higher level of their undergraduate study have significantly higher GAD-7 scores. Advanced undergraduate students often face tougher courses as well as rigid grading compared to previous years which results in a gradual increase in anxiety [77–79]. Additionally, factors such as failure in love affairs, lack of self-confidence and familial problems might contribute to increasing anxiety [80]. The results obtained from our sample also show that the GAD-7 scores are significantly higher for students from public universities. As public university students in Bangladesh mostly come from a lower socio-economic background compared to private university students, they have an additional pressure of finding jobs just after or even during their study as well as maintain good grades. As a result, fear of delayed completion of degree and uncertainty of jobs can be additional contributing factors to the high score. Our results also indicate that students who witnessed domestic violence in the family suffer more from anxiety compared to those who did not [30, 81]. Also, students who spent most of their time with family members, friends or pets, rather than on their own were significantly less anxious [81].

The GAD-7 scale is short and easily administrable and can be used effortlessly in counselling settings of academic institutions as a primary screening measure of anxiety. Hence, the validation of GAD-7 in the context of university students of Bangladesh could make a crucial contribution to the identification and treatment of mental health issues of university students.

Several limitations need to be acknowledged while interpreting the results of this study. First, the data is collected only from a relatively homogeneous sample of university students of Bangladesh in terms of socioeconomic and demographic factors. As a result, the findings should not be generalized in diverse samples. Secondly, data collection was completely web-based which can lead to participation bias and reporting bias [82]. Lastly, future research also needs to explore the sensitivity and specificity of the GAD-7 in academic settings.

This study addressed a major gap in the literature by being the first to evaluate the psychometric properties of GAD-7 in university students of Bangladesh, who are more prone to experiencing heightened anxiety. The study adds to the growing evidence of GAD-7 as a concise, simply administered self-reported questionnaire. The results also provide support for a modified unidimensional structure of GAD-7 and show high internal consistency as well as good convergent validity for the sample of university students. Such successful validation of GAD-7 scale in the context of university students of Bangladesh will allow early diagnosis and treatment of the patients, thus helping the policy makers and public health authorities to take necessary and timely interventions to deal with such disorders.

## Supporting information

S1 Table Pearson’s correlation coefficients (r) between GAD-7 items and with other questionnaires, (*n* = 677). (DOCX)

S2 Table Eigenvalue, percentage of variance, and cumulative percentage of variance for the factors derived from EFA for PHQ-9 items. (DOCX)

S3 Table Distribution of GAD-7 items. (DOCX)

S1 Dataset (DTA)

## References

[1] Bystritsky A, Khalsa SS, Cameron ME, Schiffman J. Current diagnosis and treatment of anxiety disorders. P & T: a peer-reviewed journal for formulary management [Internet]. 2013 Jan;38(1):30–57. Available from: https://pubmed.ncbi.nlm.nih.gov/23599668 23599668 PMC3628173

[2] Gonçalves DC, Pachana NA, Byrne GJ. Prevalence and correlates of generalized anxiety disorder among older adults in the Australian National Survey of Mental Health and Well-Being. Journal of Affective Disorders. 2011 Jul;132(1–2). doi: 10.1016/j.jad.2011.02.023 21429587

[3] Rapee RM. Generalized anxiety disorder: A review of clinical features and theoretical concepts. Clinical Psychology Review. 1991 Jan;11(4).

[4] Wittchen H-U, Essau CA, Krieg J-C. Anxiety Disorders: Similarities and Differences of Comorbidity in Treated and Untreated Groups. British Journal of Psychiatry. 1991 Sep 6;159(S12). 1840760

[5] Sprock J. DSM-III and DSM-III-R. In: The Encyclopedia of Clinical Psychology. Hoboken, NJ, USA: John Wiley & Sons, Inc.; 2015.

[6] American Psychiatric Association. Diagnostic and Statistical Manual of Mental Disorders. American Psychiatric Association; 2013.

[7] Dugas MJ, Anderson KG, Deschenes SS, Donegan E. Generalized anxiety disorder publications: Where do we stand a decade later? Journal of Anxiety Disorders. 2010 Oct;24(7). doi: 10.1016/j.janxdis.2010.05.012 20554425

[8] Ruscio AM, Hallion LS, Lim CCW, Aguilar-Gaxiola S, Al-Hamzawi A, Alonso J, et al. Cross-sectional Comparison of the Epidemiology of *DSM-5* Generalized Anxiety Disorder Across the Globe. JAMA Psychiatry. 2017 May 1;74(5). doi: 10.1001/jamapsychiatry.2017.0056 28297020 PMC5594751

[9] World Health Organization. Depression and Other Common Mental Disorders Global Health Estimates. 2017.

[10] Institute for Health Metrics and Evaluation (IHME). Findings from the Global Burdenof Disease Study 2017 [Internet]. Seattle, WA; 2018. Available from: www.healthdata.org

[11] Islam MS, Ferdous MZ, Potenza MN. Panic and generalized anxiety during the COVID-19 pandemic among Bangladeshi people: An online pilot survey early in the outbreak. Journal of Affective Disorders. 2020 Nov;276. doi: 10.1016/j.jad.2020.06.049 32697713 PMC7362838

[12] Islam MA, Barna SD, Raihan H, Khan MNA, Hossain MT. Depression and anxiety among university students during the COVID-19 pandemic in Bangladesh: A web-based cross-sectional survey. PLOS ONE. 2020 Aug 26;15(8). doi: 10.1371/journal.pone.0238162 32845928 PMC7449469

[13] Shovo T-E-A, Ahammed B, Khan B, Jahan N, Shohel TA, Hossain T, et al. Determinants of Generalized Anxiety, Depression, and Subjective Sleep Quality among University Students during COVID-19 Pandemic in Bangladesh. Dr Sulaiman Al Habib Medical Journal. 2021;3(1).

[14] Faisal RA, Jobe MC, Ahmed O, Sharker T. Mental Health Status, Anxiety, and Depression Levels of Bangladeshi University Students During the COVID-19 Pandemic. International Journal of Mental Health and Addiction. 2021 Jan 4; 10.1007/s11469-020-00458-y PMC7781410 33424514

[15] National Mental Health Survey in Bangladesh. NIMH Fact Sheet. 2019.

[16] Smith L, Jacob L, Yakkundi A, McDermott D, Armstrong NC, Barnett Y, et al. Correlates of symptoms of anxiety and depression and mental wellbeing associated with COVID-19: a cross-sectional study of UK-based respondents. Psychiatry Research. 2020 Sep;291. doi: 10.1016/j.psychres.2020.113138 32562931 PMC7258801

[17] Bernal-Morales B, Rodríguez-Landa JF, Pulido-Criollo F. Impact of Anxiety and Depression Symptoms on Scholar Performance in High School and University Students. In: A Fresh Look at Anxiety Disorders. InTech; 2015.

[18] Henning ER, Turk CL, Mennin DS, Fresco DM, Heimberg RG. Impairment and quality of life in individuals with generalized anxiety disorder. Depression and Anxiety. 2007;24(5). doi: 10.1002/da.20249 17091478

[19] Bourland SL, Stanley MA, Snyder AG, Novy DM, Beck JG, Averill PM, et al. Quality of life in older adults with generalized anxiety disorder. Aging & Mental Health. 2000 Nov;4(4).

[20] Barrera TL, Norton PJ. Quality of life impairment in generalized anxiety disorder, social phobia, and panic disorder. Journal of Anxiety Disorders. 2009 Dec;23(8). 10.1016/j.janxdis.2009.07.011 PMC2782397 19640675

[21] Pounds R. A review of the medical and social consequences of generalized anxiety disorder and panic disorder. The Journal of the Louisiana State Medical Society: Official Organ of the Louisiana State Medical Society. 1992 Oct 1;144(10):479–83. 1474300

[22] Spitzer RL, Kroenke K, Williams JBW, Löwe B. A Brief Measure for Assessing Generalized Anxiety Disorder. Archives of Internal Medicine. 2006 May 22;166(10). doi: 10.1001/archinte.166.10.1092 16717171

[23] Kroenke K, Spitzer RL, Williams JBW, Monahan PO, Löwe B. Anxiety Disorders in Primary Care: Prevalence, Impairment, Comorbidity, and Detection. Annals of Internal Medicine. 2007 Mar 6;146(5). 10.7326/0003-4819-146-5-200703060-00004 17339617

[24] Ruiz MA, Zamorano E, García-Campayo J, Pardo A, Freire O, Rejas J. Validity of the GAD-7 scale as an outcome measure of disability in patients with generalized anxiety disorders in primary care. Journal of Affective Disorders. 2011 Feb;128(3). doi: 10.1016/j.jad.2010.07.010 20692043

[25] Beard C, Björgvinsson T. Beyond generalized anxiety disorder: Psychometric properties of the GAD-7 in a heterogeneous psychiatric sample. Journal of Anxiety Disorders. 2014 Aug;28(6). 10.1016/j.janxdis.2014.06.002 24983795

[26] Mossman SA, Luft MJ, Schroeder HK, Varney ST, Fleck DE, Barzman DH, et al. The Generalized Anxiety Disorder 7-item scale in adolescents with generalized anxiety disorder: Signal detection and validation. Annals of clinical psychiatry: official journal of the American Academy of Clinical Psychiatrists [Internet]. 2017 Nov;29(4):227–234A. Available from: https://pubmed.ncbi.nlm.nih.gov/29069107 29069107 PMC5765270

[27] Kertz S, Bigda-Peyton J, Bjorgvinsson T. Validity of the Generalized Anxiety Disorder-7 Scale in an Acute Psychiatric Sample. Clinical Psychology & Psychotherapy. 2012 May; 10.1002/cpp.1802 22593009

[28] Delgadillo J, Payne S, Gilbody S, Godfrey C, Gore S, Jessop D, et al. Brief case finding tools for anxiety disorders: Validation of GAD-7 and GAD-2 in addictions treatment. Drug and Alcohol Dependence. 2012 Sep;125(1–2). doi: 10.1016/j.drugalcdep.2012.03.011 22480667

[29] Sinesi A, Maxwell M, O’Carroll R, Cheyne H. Anxiety scales used in pregnancy: systematic review. BJPsych Open [Internet]. 2019 Jan 10 [cited 2021 Jan 28];5(1):e5. Available from: https://www.cambridge.org/core/product/identifier/S2056472418000753/type/journal_article doi: 10.1192/bjo.2018.75 30762504 PMC6343118

[30] Zhong Q-Y, Gelaye B, Zaslavsky AM, Fann JR, Rondon MB, Sánchez SE, et al. Diagnostic Validity of the Generalized Anxiety Disorder—7 (GAD-7) among Pregnant Women. PLOS ONE. 2015 Apr 27;10(4). doi: 10.1371/journal.pone.0125096 25915929 PMC4411061

[31] Terrill AL, Hartoonian N, Beier M, Salem R, Alschuler K. The 7-Item Generalized Anxiety Disorder Scale as a Tool for Measuring Generalized Anxiety in Multiple Sclerosis. International Journal of MS Care. 2015 Mar 1;17(2). doi: 10.7224/1537-2073.2014-008 25892974 PMC4399767

[32] Loewe B, Decker O, Müller S, Brähler E, Schellberg D, Herzog W, et al. Validation and Standardization of the Generalized Anxiety Disorder Screener (GAD-7) in the General Population. Medical Care. 2008;46:266–74. doi: 10.1097/MLR.0b013e318160d093 18388841

[33] Wild B, Eckl A, Herzog W, Niehoff D, Lechner S, Maatouk I, et al. Assessing Generalized Anxiety Disorder in Elderly People Using the GAD-7 and GAD-2 Scales: Results of a Validation Study. The American Journal of Geriatric Psychiatry. 2014 Oct;22(10). doi: 10.1016/j.jagp.2013.01.076 23768681

[34] Shrestha S, Ramos K, Fletcher TL, Kraus-Schuman C, Stanley MA, Ramsey D, et al. Psychometric properties of worry and anxiety measures in a sample of african american and caucasian older adults. Aging & Mental Health. 2020 Feb 1;24(2). 10.1080/13607863.2018.1544217 30810345

[35] Hinz A, Klein AM, Brähler E, Glaesmer H, Luck T, Riedel-Heller SG, et al. Psychometric evaluation of the Generalized Anxiety Disorder Screener GAD-7, based on a large German general population sample. Journal of Affective Disorders. 2017 Mar;210. doi: 10.1016/j.jad.2016.12.012 28088111

[36] García-Campayo J, Zamorano E, Ruiz MA, Pardo A, Pérez-Páramo M, López-Gómez V, et al. Cultural adaptation into Spanish of the generalized anxiety disorder-7 (GAD-7) scale as a screening tool. Health and Quality of Life Outcomes [Internet]. 2010;8(1):8. Available from: 10.1186/1477-7525-8-8 20089179 PMC2831043

[37] Donker T, van Straten A, Marks I, Cuijpers P. Quick and easy self-rating of Generalized Anxiety Disorder: Validity of the Dutch web-based GAD-7, GAD-2 and GAD-SI. Psychiatry Research. 2011 Jun;188(1). doi: 10.1016/j.psychres.2011.01.016 21339006

[38] Sousa T V, Viveiros V, Chai M V, Vicente FL, Jesus G, Carnot MJ, et al. Reliability and validity of the Portuguese version of the Generalized Anxiety Disorder (GAD-7) scale. Health and quality of life outcomes [Internet]. 2015 Apr 25;13:50. Available from: https://pubmed.ncbi.nlm.nih.gov/25908249 doi: 10.1186/s12955-015-0244-2 25908249 PMC4424548

[39] Mills SD, Fox RS, Malcarne VL, Roesch SC, Champagne BR, Sadler GR. The psychometric properties of the Generalized Anxiety Disorder-7 Scale in Hispanic Americans with English or Spanish language preference. Cultural Diversity and Ethnic Minority Psychology. 2014 Jul;20(3). doi: 10.1037/a0036523 25045957 PMC4129392

[40] Lee B, Kim YE. The psychometric properties of the Generalized Anxiety Disorder scale (GAD-7) among Korean university students. Psychiatry and Clinical Psychopharmacology. 2019 Oct 2;29(4). doi: 10.30773/pi.2019.0226 31870089 PMC6933137

[41] Bártolo A, Monteiro S, Pereira A. Factor structure and construct validity of the Generalized Anxiety Disorder 7-item (GAD-7) among Portuguese college students. Cadernos de Saúde Pública. 2017 Sep 28;33(9). doi: 10.1590/0102-311X00212716 28977285

[42] Alghadir A, Manzar MD, Anwer S, Albougami A, Salahuddin M. Psychometric Properties of the Generalized Anxiety Disorder Scale Among Saudi University Male Students. Neuropsychiatric Disease and Treatment. 2020 Jun;Volume 16. doi: 10.2147/NDT.S246526 32606696 PMC7292258

[43] Parkerson HA, Thibodeau MA, Brandt CP, Zvolensky MJ, Asmundson GJG. Cultural-based biases of the GAD-7. Journal of Anxiety Disorders. 2015 Apr;31. doi: 10.1016/j.janxdis.2015.01.005 25725310

[44] Monteiro S, Bártolo A, Torres A, Pereira A. (Re)examining the Factorial sstructure of the Generalized Anxiety Disorder-7 in a College Students Sample. European Psychiatry. 2017 Apr 23;41(S1).

[45] Spitzer RL, Kroenke K, Williams JBW, Löwe B. A brief measure for assessing generalized anxiety disorder: The GAD-7. Archives of Internal Medicine. 2006;166(10):1092–7. doi: 10.1001/archinte.166.10.1092 16717171

[46] Omani-Samani R, Maroufizadeh S, Ghaheri A, Navid B. Generalized Anxiety Disorder-7 (GAD-7) in people with infertility: A reliability and validity study. Middle East Fertility Society Journal. 2018 Dec;23(4).

[47] Löwe B, Decker O, Müller S, Brähler E, Schellberg D, Herzog W, et al. Validation and Standardization of the Generalized Anxiety Disorder Screener (GAD-7) in the General Population. Medical Care. 2008 Mar;46(3). doi: 10.1097/MLR.0b013e318160d093 18388841

[48] Rayhan RU, Zheng Y, Uddin E, Timbol C, Adewuyi O, Baraniuk JN. Administer and Collect Medical Questionnaires with Google Documents: A Simple, Safe, and Free System [Internet]. Vol. 33, Applied Medical Informatics Original Research. 2013. Available from: www.docs.google.com. PMC3884902 24415903

[49] LightCastle Analytics Wing. Tertiary Education in Bangladesh: A Sector in Need of Reform. DATABDCO. 2019;

[50] University Grants Commission of Bangladesh. List of Public Universities [Internet]. 2020 [cited 2021 Feb 2]. Available from: http://www.ugc-universities.gov.bd/private-universities

[51] Swinson RP. The GAD-7 scale was accurate for diagnosing generalised anxiety disorder. Evidence-Based Medicine. 2006 Dec 1;11(6). doi: 10.1136/ebm.11.6.184 17213178

[52] GUZE SB. Diagnostic and Statistical Manual of Mental Disorders, 4th ed. (DSM-IV). American Journal of Psychiatry. 1995 Aug;152(8).

[53] Kroenke K, Spitzer RL, Williams JBW. The PHQ-9. Journal of General Internal Medicine. 2001 Sep;16(9). doi: 10.1046/j.1525-1497.2001.016009606.x 11556941 PMC1495268

[54] Kroenke K, Wu J, Yu Z, Bair MJ, Kean J, Stump T, et al. Patient Health Questionnaire Anxiety and Depression Scale. Psychosomatic Medicine. 2016;78(6). doi: 10.1097/PSY.0000000000000322 27187854 PMC4927366

[55] Pituch KA, Stevens J (James P. Applied multivariate statistics for the social sciences: analyses with SAS and IBM’s SPSS. Vol. Sixth Edition. Routledge; 2016.

[56] Flora DB, Curran PJ. An Empirical Evaluation of Alternative Methods of Estimation for Confirmatory Factor Analysis With Ordinal Data. Psychological Methods. 2004 Dec;9(4). doi: 10.1037/1082-989X.9.4.466 15598100 PMC3153362

[57] Browne MW, Cudeck R. Alternative Ways of Assessing Model Fit. Sociological Methods & Research. 1992 Nov 29;21(2).

[58] Steiger JH. Structural Model Evaluation and Modification: An Interval Estimation Approach. Multivariate Behavioral Research. 1990 Apr;25(2). doi: 10.1207/s15327906mbr2502_4 26794479

[59] Fan X, Sivo SA. Sensitivity of Fit Indices to Model Misspecification and Model Types. Multivariate Behavioral Research. 2007 Oct 10;42(3).

[60] Tucker LR, Lewis C. A reliability coefficient for maximum likelihood factor analysis. Psychometrika. 1973 Mar;38(1).

[61] Zijlmans EAO, Tijmstra J, van der Ark LA, Sijtsma K. Item-Score Reliability in Empirical-Data Sets and Its Relationship With Other Item Indices. Educational and Psychological Measurement. 2018 Dec 27;78(6). doi: 10.1177/0013164417728358 30542214 PMC6236637

[62] Lord FM, Novick MR, Birnbaum A. Statistical theories of mental test scores. Statistical theories of mental test scores. Oxford, England: Addison-Wesley; 1968.

[63] Thorndike RM. Book Review: Psychometric Theory (3rd ed.) by Jum Nunnally and Ira Bernstein New York: McGraw-Hill, 1994, xxiv + 752 pp. Applied Psychological Measurement. 1995 Sep 26;19(3).

[64] Dodge Yadolah. The Concise Encyclopedia of Statistics. New York, NY: Springer New York; 2008.

[65] Brown TA. Confirmatory factor analysis for applied research. Guilford publications; 2015.

[66] Rutter LA, Brown TA. Psychometric Properties of the Generalized Anxiety Disorder Scale-7 (GAD-7) in Outpatients with Anxiety and Mood Disorders. Journal of Psychopathology and Behavioral Assessment. 2017 Mar 10;39(1). doi: 10.1007/s10862-016-9571-9 28260835 PMC5333929

[67] Hettema JM. The nosologic relationship between generalized anxiety disorder and major depression. Depression and Anxiety. 2008 Apr 1;25(4). doi: 10.1002/da.20491 18412057

[68] Schoevers RA. Depression and Generalized Anxiety Disorder: Co-Occurrence and Longitudinal Patterns in Elderly Patients. American Journal of Geriatric Psychiatry. 2005 Jan 1;13(1). doi: 10.1176/appi.ajgp.13.1.31 15653938

[69] Ruscio AM, Chiu WT, Roy-Byrne P, Stang PE, Stein DJ, Wittchen H-U, et al. Broadening the definition of generalized anxiety disorder: Effects on prevalence and associations with other disorders in the National Comorbidity Survey Replication. Journal of Anxiety Disorders. 2007 Jan;21(5). doi: 10.1016/j.janxdis.2006.10.004 17118626 PMC2475335

[70] Moreno E, Muñoz-Navarro R, Medrano LA, González-Blanch C, Ruiz-Rodríguez P, Limonero JT, et al. Factorial invariance of a computerized version of the GAD-7 across various demographic groups and over time in primary care patients. Journal of Affective Disorders. 2019 Jun;252. doi: 10.1016/j.jad.2019.04.032 30981054

[71] Fu W, Yan S, Zong Q, Anderson-Luxford D, Song X, Lv Z, et al. Mental health of college students during the COVID-19 epidemic in China. Journal of Affective Disorders. 2021 Feb;280. doi: 10.1016/j.jad.2020.11.032 33197782 PMC7656159

[72] Eller T, Aluoja A, Vasar V, Veldi M. Symptoms of anxiety and depression in Estonian medical students with sleep problems. Depression and Anxiety. 2006;23(4). doi: 10.1002/da.20166 16555263

[73] Mikolajczyk RT, Maxwell AE, El Ansari W, Naydenova V, Stock C, Ilieva S, et al. Prevalence of depressive symptoms in university students from Germany, Denmark, Poland and Bulgaria. Social Psychiatry and Psychiatric Epidemiology. 2008 Feb 23;43(2). 10.1007/s00127-007-0282-0 18038173

[74] Vesga Lopez O, Schneier F, Wang S, Heimberg RG, Liu SM, Hasin DS, et al. Gender Differences in Generalized Anxiety Disorder: Results From the National Epidemiologic Survey on Alcohol and Related Conditions (NESARC). The Journal of clinical psychiatry. 2008 Nov 18; PMC4765378 19192444

[75] Bahrami F, Yousefi N. Females are more anxious than males: a metacognitive perspective. Iranian journal of psychiatry and behavioral sciences [Internet]. 2011;5(2):83–90. Available from: https://pubmed.ncbi.nlm.nih.gov/24644451 24644451 PMC3939970

[76] Hossain S, Anjum A, Uddin ME, Rahman MA, Hossain MF. Impacts of socio-cultural environment and lifestyle factors on the psychological health of university students in Bangladesh: A longitudinal study. Journal of Affective Disorders. 2019 Sep;256. doi: 10.1016/j.jad.2019.06.001 31226611

[77] Sakin Ozen N, Ercan I, Irgil E, Sigirli D. Anxiety Prevalence and Affecting Factors Among University Students. Asia Pacific Journal of Public Health. 2010 Jan 23;22(1). 10.1177/1010539509352803 20032042

[78] Hyseni Duraku Z. Factors Influencing Test Anxiety among University Students. The European Journal of Social and Behavioural Sciences. 2017 Jan 1;1(1).

[79] Vitasari P, Wahab MNA, Othman A, Herawan T, Sinnadurai SK. The Relationship between Study Anxiety and Academic Performance among Engineering Students. Procedia—Social and Behavioral Sciences. 2010;8.

[80] Mamun MA, Hossain MS, Griffiths MD. Mental Health Problems and Associated Predictors Among Bangladeshi Students. International Journal of Mental Health and Addiction. 2019 Oct 29;

[81] Kader Maideen SF, Mohd Sidik S, Rampal L, Mukhtar F. Prevalence, associated factors and predictors of anxiety: a community survey in Selangor, Malaysia. BMC Psychiatry. 2015 Dec 24;15(1). 10.1186/s12888-015-0648-x PMC4620008 26497745

[82] Heiervang E, Goodman R. Advantages and limitations of web-based surveys: evidence from a child mental health survey. Social Psychiatry and Psychiatric Epidemiology. 2011 Jan 18;46(1). doi: 10.1007/s00127-009-0171-9 19921078

