# Supplementary material for: Validity and reliability of the Generalized Anxiety Disorder-7 (GAD-7) among university students of Bangladesh
Source: PLoS One. 2021 Dec 16;16(12):e0261590. doi: 10.1371/journal.pone.0261590 (PMC8675645; doi:10.1371/journal.pone.0261590)
Supplement: S1 Table — (DOCX) [file pone.0261590.s001.docx]

|  | | **GAD-7** | |  |  |  |  |  |  |
| --- | --- | --- | --- | --- | --- | --- | --- | --- | --- |
| **PHQ-9** | | 0.751 | |  |  |  |  |  |  |
| **PHQ-ADS** | | 0.934 | |  |  |  |  |  |  |
|  | **GAD-1** | | **GAD-2** | | **GAD-3** | **GAD-4** | **GAD-5** | **GAD-6** | **GAD-7** |
| **GAD-1** | 1.000 | |  | |  |  |  |  |  |
| **GAD-2** | 0.697 | | 1.000 | |  |  |  |  |  |
| **GAD-3** | 0.666 | | 0.731 | | 1.000 |  |  |  |  |
| **GAD-4** | 0.512 | | 0.572 | | 0.603 | 1.000 |  |  |  |
| **GAD-5** | 0.433 | | 0.483 | | 0.495 | 0.498 | 1.000 |  |  |
| **GAD-6** | 0.533 | | 0.547 | | 0.578 | 0.476 | 0.481 | 1.000 |  |
| **GAD-7** | 0.582 | | 0.566 | | 0.575 | 0.486 | 0.445 | 0.528 | 1.000 |

**S1 Table: Pearson’s correlation coefficients (r) between GAD-7 items and with other questionnaires, (*n*= 677)**

PHQ-9= Patient Health Questionnaire-9, GAD-7= Generalized Anxiety Disorder-7, PHQ-ADS= Patient Health Questionnaire Anxiety and Depression Scale
All values were significant at *p*<0.01
