# Supplementary material for: Validity and reliability of the Generalized Anxiety Disorder-7 (GAD-7) among university students of Bangladesh
Source: PLoS One. 2021 Dec 16;16(12):e0261590. doi: 10.1371/journal.pone.0261590 (PMC8675645; doi:10.1371/journal.pone.0261590)
Supplement: S2 Table — (DOCX) [file pone.0261590.s002.docx]

| **Factor** | **Eigenvalue** | **Percentage of variance** | **Cumulative percentage of variance** |
| --- | --- | --- | --- |
| Factor 1 | 4.300 | 0.614 | 0.614 |
| Factor 2 | 0.650 | 0.093 | 0.707 |
| Factor 3 | 0.546 | 0.078 | 0.785 |
| Factor 4 | 0.467 | 0.067 | 0.852 |
| Factor 5 | 0.454 | 0.065 | 0.917 |
| Factor 6 | 0.321 | 0.046 | 0.963 |
| Factor 7 | 0.262 | 0.037 | 1.000 |

**S2 Table: Eigenvalue, percentage of variance, and cumulative percentage of variance for the factors derived from EFA for PHQ-9 items**
