# Supplementary material for: Validity and reliability of the Generalized Anxiety Disorder-7 (GAD-7) among university students of Bangladesh
Source: PLoS One. 2021 Dec 16;16(12):e0261590. doi: 10.1371/journal.pone.0261590 (PMC8675645; doi:10.1371/journal.pone.0261590)
Supplement: S3 Table — (DOCX) [file pone.0261590.s003.docx]

| **GAD-7 items** | **Not at all**  **(%)** | **Several days**  **(%)** | **More than half the days**  **(%)** | **Nearly everyday**  **(%)** |
| --- | --- | --- | --- | --- |
| 1. Feeling nervous, anxious or on edge? | 22.90 | 35.01 | 19.05 | 23.04 |
| 2. Not being able to stop or control worrying? | 23.93 | 28.80 | 18.02 | 29.25 |
| 3. Worrying too much about different things? | 19.20 | 28.51 | 20.38 | 31.91 |
| 4. Trouble relaxing? | 28.36 | 33.53 | 17.87 | 20.24 |
| 5. Being so restless that it is hard to sit still? | 40.77 | 28.95 | 15.81 | 14.48 |
| 6. Becoming easily annoyed or irritable? | 25.55 | 31.91 | 21.12 | 21.42 |
| 7. Feeling afraid as if something awful might happen? | 22.60 | 30.72 | 15.95 | 30.72 |

**S3 Table: Distribution of GAD-7 items**
